# pH-Sensitive Chitosan Nanoparticles for Salivary Protein Delivery

**DOI:** 10.3390/nano11041028

**Published:** 2021-04-17

**Authors:** Yi Zhu, Lina M. Marin, Yizhi Xiao, Elizabeth R. Gillies, Walter L. Siqueira

**Affiliations:** 1 School of Biomedical Engineering, The University of Western Ontario, 1151 Richmond Street, London, ON N6A 3K7, Canada; yzhu336@uwo.ca (Y.Z.); egillie@uwo.ca (E.R.G.); 2 College of Dentistry, University of Saskatchewan, 105 Wiggins Rd, Saskatoon, SK S7N 5E4, Canada; lina.marin@usask.ca; 3 Schulich Medicine and Dentistry, The University of Western Ontario, 1151 Richmond Street, London, ON N6A 5C1, Canada; yxiao32@uwo.ca; 4 Department of Chemistry, Department of Chemical and Biochemical Engineering, The University of Western Ontario, 1151 Richmond Street, London, ON N6A 5B7, Canada

**Keywords:** protein carrier, drug delivery, polymeric nanoparticles, biopolymers

## Abstract

Salivary proteins such as histatins (HTNs) have demonstrated critical biological functions directly related to tooth homeostasis and prevention of dental caries. However, HTNs are susceptible to the high proteolytic activities in the oral environment. Therefore, pH-sensitive chitosan nanoparticles (CNs) have been proposed as potential carriers to protect proteins from enzymatic degradation at physiological salivary pH. Four different types of chitosan polymers were investigated and the optimal formulation had good batch to batch reproducibility, with an average hydrodynamic diameter of 144 ± 6 nm, a polydispersity index of 0.15 ± 0.04, and a zeta potential of 18 ± 4 mV at a final pH of 6.3. HTN3 encapsulation and release profiles were characterized by cationic polyacrylamide gel electrophoresis. The CNs successfully encapsulated HTN3 and selectively swelled at acidic pH to facilitate HTN3 release. Protection of HTN3 against enzymatic degradation was investigated in diluted whole saliva. HTN3 encapsulated in the CNs had a prolonged survival time compared to the free HTN3. CNs with and without HTN3 also successfully reduced biofilm weight and bacterial viability. The results of this study have demonstrated the suitability of CNs as potential protein carriers for oral applications, especially for complications occurring at acidic conditions.

## 1. Introduction

Saliva is a complex fluid composed of proteins, enzymes, and a variety of electrolytes. Many physiological functions, such as chewing, digestion, wetting, and lubrication are regulated by saliva [1]. The concentration of proteins present in the saliva may be used to monitor oral health, as the severity and occurrence of oral diseases have been associated with the composition and quantitative changes in salivary proteins [2]. Many of the salivary proteins are active in the regulation of tooth homeostasis, either by directly exerting antimicrobial effects or interfering with microbial colonization [3]. Examples include statherin, histatins (HTNs), defensins, lactoferrin, and mucin [4]. In particular, the HTNs are of great interest because these proteins are multifunctional. They have demonstrated biological functions including the inhibition of calcium and phosphate precipitation on enamel and antimicrobial activities, which are directly related to the regulation of oral homeostasis [5,6,7]. The HTNs mainly consist of HTN1, HTN3, and HTN5, and each supplies about 20–30% of the total HTN pool [8]. HTN1 is the only phosphorylated HTN able to reduce bacterial colonization on tooth surfaces [9]. HTN5 has the most potent antifungal activity against the pathogenic yeast *Candida albicans* [10]. HTN3 was demonstrated to be the most effective in killing against *Streptococcus mutans* (*S. mutans*) [11], which is a significant contributor towards dental caries.

These proteins are often present in low concentrations inside the oral cavity due to the high proteolytic activity of saliva [12,13]. Therefore, we proposed a delivery system to encapsulate and protect these proteins for their use as inhibitors of tooth decay. The use of stimulus-responsive polymeric nanoparticles synthesized from natural polymers has recently gained substantial attention, as such smart delivery systems have the potential to improve the overall colloidal stability of the encapsulated molecules and modulate pharmacokinetics, often resulting in reduced toxicity and enhanced efficacy [14,15,16,17]. The encapsulated cargo can be released in a stimulus-responsive manner. Examples of stimuli include changes in pH, ionic strength, temperature, UV light, magnetic field, or the presence of specific biological molecules [18,19,20,21]. In particular, for delivering drugs to the oral cavity, it would be ideal for the delivery system to remain stable at physiological salivary pH and selectively release the encapsulated cargo under acidic conditions. The pH-responsive property is crucial because the oral environment acidifies following carbohydrate intake as a result of the consumption of food and beverages. These conditions promote the development of oral diseases such as dental caries and dental erosion [22,23].

Many polymer-based materials have been studied for applications in controlled drug delivery, including naturally occurring biopolymers. For instance, chitosan is a copolymer composed of *N*-acetyl-d-glucosamine and β(1–4) linked d-glucosamine [24]. It is mainly obtained through the deacetylation of chitin under alkaline chemicals [25]. Chitin is a biopolymer found in the exoskeletons of crustaceans, insects, and some fungi [26]. As the only known naturally occurring polycationic polysaccharide and with its ability to interact with anionic molecules, chitosan and its derivatives have been studied extensively for applications in the agricultural, medicinal, and pharmaceutical industries [27,28,29].

Previous studies have utilized chitosan in toothpaste as an antimicrobial agent due to its broad antimicrobial spectrum, covering both Gram-negative and Gram-positive bacteria and fungi [30,31]. Aside from its antimicrobial effect, chitosan also offers other advantages including biodegradability [32] and biocompatibility [33]. It also exhibits pH-dependent solubility due to the presence of amino groups on the polymer chains [34]. The functional amino groups can serve as platforms for interactions with other anionic molecules, where the choice of anionic molecules depends on the intended applications.

Chitosan nanoparticles (CNs) have been commonly used to deliver therapeutic agents, such as insulin [35], tretinoin [36], and doxorubicin [37]. In the current work, to the best of our knowledge, for the first time we propose pH-sensitive chitosan nanoparticles for salivary protein delivery. We hypothesize that salivary protein-loaded CNs can selectively release the encapsulated proteins under low pH environments, offer protection against proteolysis at the physiological salivary pH, and reduce *S. mutans* biofilm formation on hydroxyapatite. To test these hypotheses, HTN3 was selected as the target protein, and our objectives were to: (1) optimize CN formulation via ionic gelation with four different types of chitosan polymers; (2) characterize both blank and HTN3-loaded CNs; (3) quantify encapsulation and release profiles of HTN3; (4) assess the protection offered by encapsulation against enzymatic degradation in human saliva; and (5) evaluate the effectiveness of HTN3-loaded CNs in reducing *S. mutans* biofilm formation. 

## 2. Materials and Methods

### 2.1. Materials

Ultra-low molecular weight (MW) chitosan (20 kg/mol, 91% deacetylated) and low MW chitosan (1) (250 kg/mol, 91% deacetylated) were purchased from Glentham Life Sciences (Corsham, UK). The other low MW chitosan (2) (50–190 kg/mol, 75–85% deacetylated), medium MW chitosan (190–310 kg/mol, 75–85% deacetylated), and ZipTip C18 pipette tips were acquired from Millipore Sigma (Oakville, ON, Canada). All filters, including 0.45 and 0.22 µm syringe filters and 10k Nanosep filters were purchased from Pall Corporation (Mississauga, ON, Canada). HTN3 was acquired from Synpeptide Co., Ltd. (Shanghai, China). *S. mutans* UA159 was kindly donated by Dr. Yoav Finer from the University of Toronto (Toronto, ON, Canada). Ceramic hydroxyapatite discs with 5 mm diameter and 2 mm thickness were obtained from Clarkson Chromatography Products Inc. (South Williamsport, PA, USA). Bicinchoninic acid assay kit was acquired from Thermo Fisher Scientific (Mississauga, ON, Canada). All other chemicals, including sodium tripolyphosphate (TPP), were purchased from Millipore Sigma (Oakville, ON, Canada).

### 2.2. Optimized CNs Preparation

The ionic gelation procedure described previously was modified to optimize CNs synthesis [38]. Briefly, preliminary experiments were done to assess the effect of chitosan molar mass and degree of deacetylation, pH of chitosan/TPP solutions, and chitosan to TPP mass ratios on CN characteristics. These particle preparations were performed in triplicate. After determining the optimal conditions, unloaded CNs were synthesized by dissolving 100 mg of ultra-low MW chitosan in 100 mL of 0.4% *v/v* concentrated HCl. The pH of the chitosan solution was adjusted to 5.9 and the solution was filtered through a 0.45 µm syringe filter. Then, 100 mg of TPP was dissolved in 100 mL of Milli-Q water, its pH was adjusted to 6.0 and solution was filtered through a 0.22 µm syringe filter. Finally, 0.36 mL of TPP solution was added dropwise into 2.5 mL of chitosan solution under constant stirring at 700 rpm, such that the mass ratio of chitosan to TPP was 6.94:1. HTN3-loaded CNs were synthesized by mixing HTN3 with the chitosan solution before ionic gelation with TPP. Briefly, 0.29 mL of the 0.1% *w/v* TPP solution was introduced dropwise into 2 mL of 0.1% *w/v* chitosan and 0.0025% *w/v* HTN3 under constant stirring at 700 rpm. The loading ratio of chitosan to HTN3 by mass was 40:1, and 0.45 mL of the suspension contained 10 μg of HTN3. The final pH of the nanoparticle solution was 6.3.

### 2.3. Characterizations of CNs

Unloaded CNs and HTN3-loaded CNs were initially characterized by measuring the Z-average particle diameter, polydispersity index (PDI), and zeta potential by dynamic light scattering (DLS, Malvern Zetasizer Nano ZS instrument, Malvern Instruments Ltd, Malvern, UK) at a wavelength of 630 nm and a constant temperature of 25 °C. To determine the morphology of CNs, an aliquot from each CNs suspension was firstly desalted by dialyzing it against 1 L of deionized water for 3 h under constant stirring, using a 50 kg/mol MW cut-off regenerated cellulose dialysis tubing (Spectrum Labs, New Brunswick, NJ, USA). Then, samples were placed onto Formvar^®^-coated copper grid, dried overnight under an air atmosphere, and the morphology of the CNs was visualized by transmission electron microscopy (TEM, Philips CM10, Philips, Amsterdam, The Netherlands) at 80 kV. To investigate the pH-dependence of CN size, initial (time = 0 min) particle size measurements were taken at a starting pH of 6.3. The pH of the suspension was then adjusted to 3.0, 4.0, or 5.0. After the pH adjustments, particle size, PDI, and zeta potential were assessed by DLS at 10, 20, 30, 60, 120, and 240 min. To evaluate colloidal stability, CNs were stored at 4 °C for 61 days. Particle size and zeta potential were measured by DLS on days 1, 10, 15, 22, 30, 35, 45, 52, and 61.

### 2.4. Encapsulation and Release of HTN3

To assess the pH-dependent release of HTN3 from HTN3-loaded CNs, 0.05 mL of buffer (25 mM phosphate buffer/500 mM NaCl solution, pH 6.8), was added to 0.45 mL of HTN3-loaded CNs suspension. The 0.5 mL of suspension was then filtered with a 10 kDa cut-off Nanosep filter by centrifuging the suspension at 14,000× *g* for 10 min to separate free HTN3 (4063 Dalton) from the encapsulated CNs. The filtrate was collected to determine encapsulation efficiency by cationic polyacrylamide gel electrophoresis (cationic-PAGE) [39]. The pH of the retentate on top of the filter was adjusted to pH 3.0, 4.0, or 5.0 and incubated at 37 °C for 30 min. The suspension was centrifuged again, allowing the passage of released HTN3 through the filter. Subsequently, the filtrate was collected to determine the extent of protein release by cationic-PAGE, using pure HTN3 as a reference control. HTN3 encapsulation efficiency was tested for loading ratios of 2%, 5%, and 10% *w/w* (HTN3 to chitosan). Encapsulation samples were prepared as previously described with modifications to the mass ratio of HTN3 to chitosan. The encapsulation efficiency was determined by Image Lab (BioRad) via the relative quantity tool, corresponding to the ratio of the band volume and intensity divided by the reference (HTN3 standard) band volume and intensity. To evaluate the cumulative release of HTN3, HTN3-loaded CNs were prepared as previously described. Then, the retentate on top of the filter was incubated for 30, 60, 90, 120, 150, 180, and 210 min at 37 °C in buffer solutions with different pH values (6.8, 3.0, 4.0, or 5.0). Then, samples were centrifuged at 14,000× *g* for 10 min to separate the released protein from the CNs. Subsequently, the filtrate was collected. This procedure was repeated for each time point and the filtrate and a HTN3 standard were analyzed by cationic-PAGE as previously described. The ability of the HTN3-loaded CNs to release HTN3 under pH-cycling conditions, reflecting the pH changes that occur in the oral cavity, was determined by incubating the CNs in buffer with pH of 6.8 and then in buffer with pH 4.0. This cycle was repeated for a total of four times, and the amount of HTN3 released at each pH in function of time was determined by cationic-PAGE, following the protocol previously described.

### 2.5. Protein Degradation Study

Considering that proteins are susceptible to proteolytic degradation inside the oral cavity [40], the protection offered by encapsulation was evaluated in diluted whole saliva. Stimulated whole saliva was collected by chewing a 5 cm × 5 cm parafilm piece from three healthy individuals, between 9:00 am to 11:00 am, to minimize the effect of circadian rhythm [13], and at least two hours after breakfast. Whole saliva was kept on ice during the collection and then was immediately centrifuged for 10 min 14,000× *g* at 4 °C to separate the bacteria, cells, and other debris from the supernatant containing salivary proteins. The concentration of salivary proteins in the supernatant, referred as whole saliva supernatant (WSS), was quantified using a bicinchoninic acid assay kit. The degradation of free HTN3 was assessed in 10-fold diluted WSS, following the protocol previously reported [6]. Briefly, 50 μg of free HTN3 was added to diluted WSS (100 μg salivary protein/mL) in a final volume of 1 mL. The pH was adjusted to 6.8 using 1 M HCl or 1 M NaOH, depending on the pH of the WSS. Then, an aliquot of 100 μL was incubated for 0, 0.5, 1, 2, 3, or 6 h at 37 °C. At each time point, samples were boiled for 5 min to terminate proteolytic activity, dried, and desalted. The extent of degradation was quantified after cationic-PAGE, using Image Lab relative quantity tool. Subsequently, the degradation of HTN3-loaded CNs in WSS was assessed by mixing 0.5 mL of the HTN3-loaded CNs (containing 50 μg of HTN3) with diluted WSS (100 μg salivary protein/mL) to reach a final volume of 1 mL. The final pH was standardized to 6.8 with 1 M HCl or 1 M NaOH, depending on the initial pH of saliva. An aliquot of 100 μL was collected after incubation at 37 °C at the following time points: 0, 0.5, 1, 2, 3, and 6 h. The pH was adjusted to 3 to release all of the HTN3 within the delivery system. After centrifugation with a 10 kDa Nanosep filter, the filtrate was retrieved and boiled for 5 min to terminate proteolytic activity. Samples were dried and desalted, followed by cationic-PAGE. The extent of degradation was quantified by Image Lab through the relative quantity tool.

### 2.6. Streptococcus mutans Killing Assay and Biofilm Formation

*S. mutans* (UA 159) colonies were spiked from blood agar and grown for 14 h in 10 mL of tryptone yeast extract broth (TYEB) supplemented with 1% glucose at 37 °C and 10% CO_2_. After incubation, the bacterial suspension was washed twice with 0.9% NaCl and the pellet was resuspended in 1.2 mL of phosphate-buffered saline (PBS). The optical density (OD) at 600 nm was adjusted to 1.5, corresponding to a bacterial concentration of 1 × 10^9^ CFU/mL, based on a growth curve previously done. An aliquot of 0.1 mL of the bacterial suspension with 1.5 OD reading was added to 9.9 mL of PBS to obtain a bacterial concentration of 10^7^ CFU/mL. This suspension was then added to an equal volume of a serial dilution series of HTN3 from 0 to 200 μM in a 96-well polypropylene microtiter plate. The samples were then incubated at 37 °C for 1.5 h. After incubation, each sample was diluted 10^3^ and 10^4^-fold in PBS, and 20 μL of each dilution was plated onto Todd Hewitt broth (THB) agar plates. Bacterial viability was assessed by colony counting using comparisons against control samples incubated without HTN3. The IC50 value was calculated based on the dose–response curve and used in the biofilm study described below. To assess the capability of HTN3-loaded CNs to reduce *S. mutans* biofilm formation on hydroxyapatite (HA) discs, the discs were fixated on the interior of a 24-well polypropylene microtiter plate lid, where the discs were carefully positioned so that the plate lid fits both 24 and 96-well plates. The plate lid was placed to immerse all discs in a 96-well plate filled with 200 μL of 5 different treatment solutions (*n* = 6/group): control (PBS), 7.4 μM HTN3, 0.1% *w/v* unloaded CNs, 0.1% *w/v* CNs containing 7.4 μM of encapsulated HTN3, and 12,300 ppm fluoride solution. Treatments were done under constant stirring for 2 h at 37 °C to allow the formation of a single-component pellicle onto HA, simulating the formation of the acquired enamel pellicle in the oral cavity. Subsequently, the plate cover was placed on a 24-well plate with 2 mL of 0.9% *w/v* NaCl to wash the discs. Afterwards the discs were immersed into 2 mL of TYEB supplemented with 1% *w/v* glucose and 10^7^ CFU/mL of bacteria. The discs were then incubated at 37 °C in 10% CO_2_ for 8 h. After incubation, the discs were washed again with NaCl, followed by further incubation for 16 h immersed in 2 mL of TYEB supplemented with 0.1 mM glucose. Over the next 4 days, the discs were incubated in a repeating cycle between solution 1 and 2 where solution 1 was TYEB supplemented with 1% *w/v* sucrose for 8 h, and solution 2 was TYEB supplemented with 0.1 mM glucose for 16 h. On the 6th day, all discs were transferred to separate tubes filled with 1 mL of PBS, followed by sonication to remove the biofilms off the discs. An aliquot of 0.5 mL of the suspension was transferred to preweighted tubes and centrifuged at 14,000× *g* for 5 min. The supernatant was removed, and the biofilm wet weight was measured. The same suspension was diluted 10^6^ and 10^7^ times in PBS, and 20 μL of each dilution was plated onto THB agar plates, incubated for 48 h at 37 °C and 10% CO_2_, and then bacterial viability was assessed by colony counting.

### 2.7. Statistical Analyses

Statistical analyses were performed using software Prism 8.0 GraphPad. Biofilm mass and bacterial viability were analyzed by ordinary one-way ANOVA followed by Tukey’s multiple comparisons test between each treatment group. The level of significance (α) was set at 0.05 (95% confidence interval).

## 3. Results

### 3.1. Results

#### 3.1.1. Optimization of CNs Formulation

**Figure 1:**
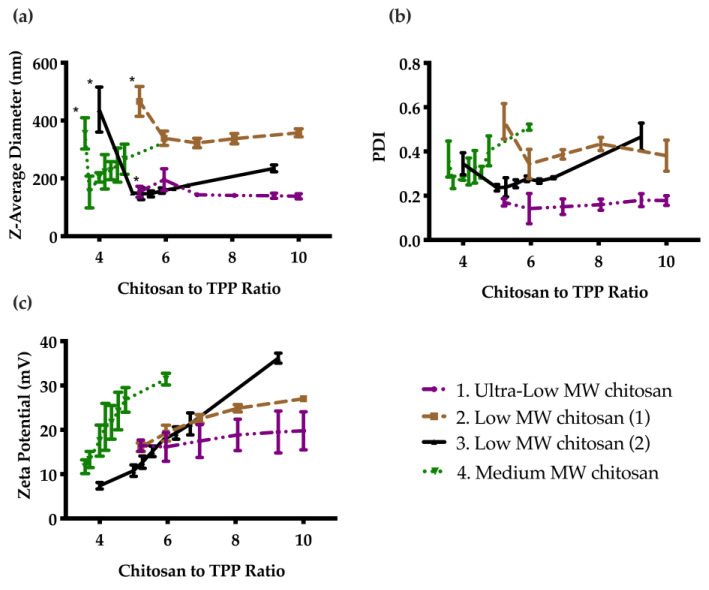
Effect of chitosan to TPP mass ratio on particle properties as measured by DLS: (**a**) Z-average diameter; (**b**) PDI; and (**c**) Zeta potential. Error bars correspond to the standard deviations on triplicate particle preparations. The particles were unstable and sedimented below a certain ratio (indicated by *), then the diameter generally decreased as the ratio was increased, and finally increased as the ratio was further increased. PDI followed a similar trend, while the zeta potential continually increased due to the increasing cationic charge with increasing chitosan to TPP ratio.

Four different types of chitosan were investigated to select the best formulation. As shown in Figure 1a, in general the particles were unstable and aggregated at low chitosan to TPP mass ratios. At higher ratios, stable particles were formed and the diameter decreased. Upon further increasing the chitosan to TPP mass ratio, the particle diameter steadily increased for most systems. PDI followed a similar trend as demonstrated in Figure 1b. Zeta potential increased steadily with a corresponding increase in chitosan to TPP ratio. Based on these results, CNs prepared from ultra low MW chitosan were selected as the optimal formulation, because they had the smallest particle diameter (Figure S1), lowest PDI, with a fairly high zeta potential. The particle diameter, PDI, and zeta potential were stable at 4 °C for at least 60 days (Figure S2).

#### 3.1.2. TEM Images of Unloaded and HTN3-Loaded CNs

**Figure 2:**
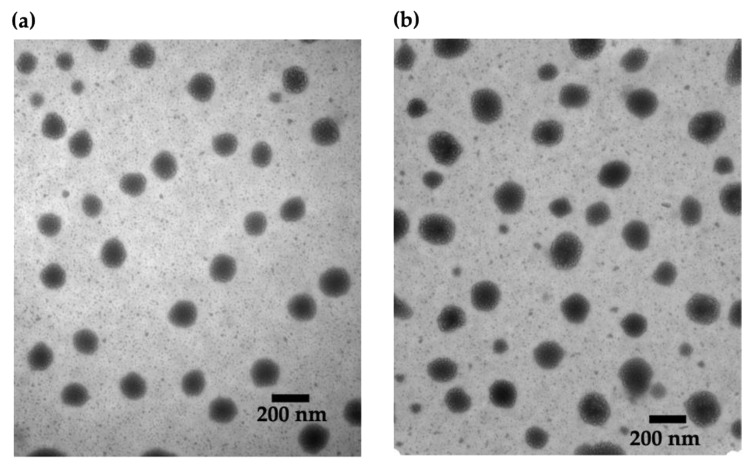
TEM images of CNs: (**a**) unloaded and (**b**) HTN3-loaded at a 2% *w/w* loading ratio. There are no obvious differences between the samples.

The selected ultra-low CNs were then visualized with TEM. As shown in Figure 2, the CNs had a spherical morphology, and the observed diameters were in good agreement with the data obtained from DLS. There were no obvious differences in size or dispersity among unloaded CNs and those with different loading ratios (Figure S3). 

#### 3.1.3. pH-Dependent Swelling CNs

**Figure 3:**
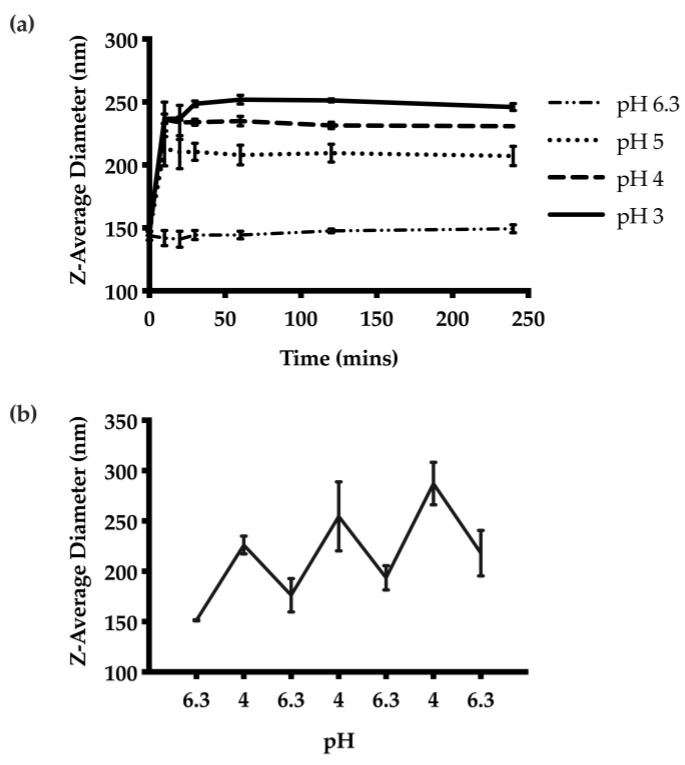
pH-responsive behaviour of the CNs: (**a**) Z-average diameters over time at different pH values from 3 to 6.3. The results suggest the degree of swelling is proportional to the acidity of the suspension. The swelling behaviour was also rapid, and reached equilibrium 10 min after pH adjustment. (**b**) The pH-responsive behaviour was further examined through pH cycling between pH 6.3 and 4. The nanoparticles were able to swell and contract accordingly based on the pH.

The pH-responsive properties of the unloaded CNs were examined first by adjusting the pH from the initial pH of 6.3 for the formulation to pH 3, 4, or 5. DLS measurements of the Z-average diameters at each pH indicated greater swelling at lower pH values (Figure 3a). The CNs swelled from 146 nm at pH 6.3 to 260 nm at pH 3. The swelling was rapid at each pH, reaching an equilibrium diameter at 10 min. We also subjected the CNs suspension to pH-cycling between 6.3 and 4. Measurements of the Z-average diameter suggested that the CNs were able to selectively swell under acidic conditions and then to contract when the pH was increased upon removal of the stimulus (Figure 3b). However, there was a general trend towards larger diameters with repeated swelling cycles.

#### 3.1.4. pH-Dependent Release of HTN3 from CNs

**Figure 4:**
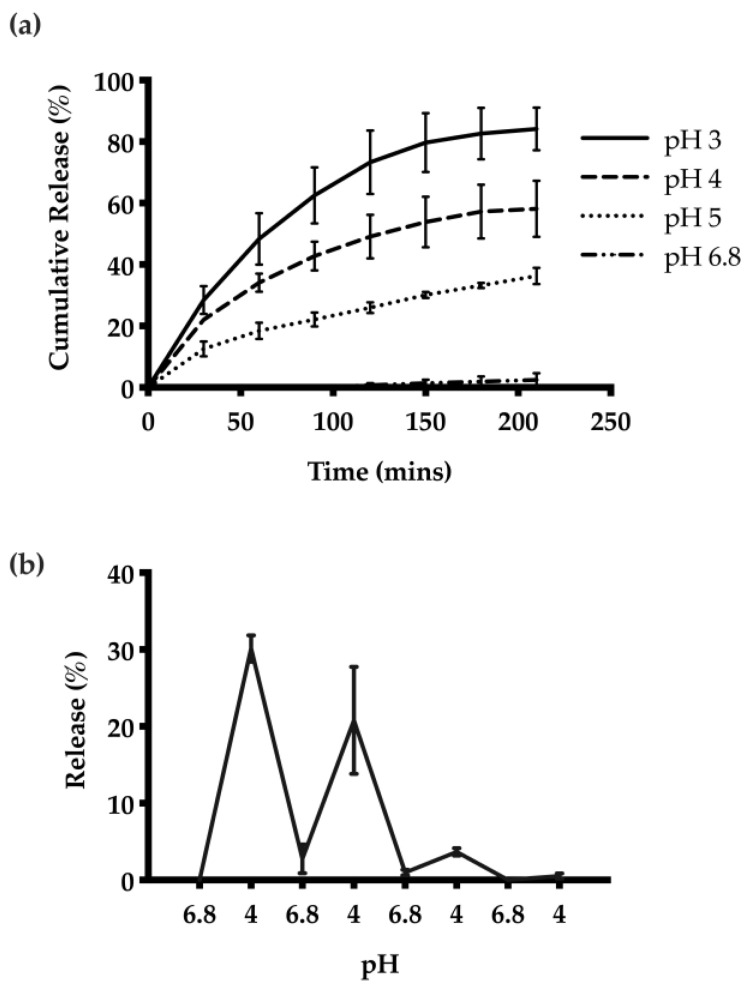
(**a**) Release of HTN3 from CNs at different pH values. The highest extent of release and most rapid release was observed at pH 3, and there was minimal release at pH 6.8. On average, at pH 3, the CNs were able to release 84% ± 7% of the encapsulated protein, 58% ± 9% at pH 4, 36% ± 3% at pH 5, and 2% ± 2% at pH 6.8. (**b**) pH cycling release results show that the CNs can adapt to environmental pH changes and can release protein selectively at pH 4 over 3 pH cycles.

The release kinetics of HTN3 encapsulated CNs were investigated at different pH values. The highest cumulative amount of HTN3 release was observed at pH 3, and there was minimal release at pH 6.8. On average, at pH 3, the CNs were able to release 84% ± 7% of the encapsulated HTN3, 58% ± 9% at pH 4, 36% ± 3% at pH 5, and 2% ± 2% at pH 6.8 over seven separate releases (Figure 4a). Thus, the CNs were able to respond to the environmental pH changes and release the protein selectively at acidic pH. pH-cycling was also performed to reflect the pH changes that happen many times in the oral cavity throughout the day. HTN3 loaded CNs were subjected to pH treatments in the following sequence: 6.8, 4, 6.8, 4, 6.8, 4, 6.8, and 4. After isolation of released protein and cationic-PAGE at each pH change, protein bands of HTN3 were only observed at pH 4 and were absent at pH 6.8 (Figure 4b). This result shows the pH selective release for the CNs.

#### 3.1.5. HTN3 Degradation Study in WSS

**Figure 5:**
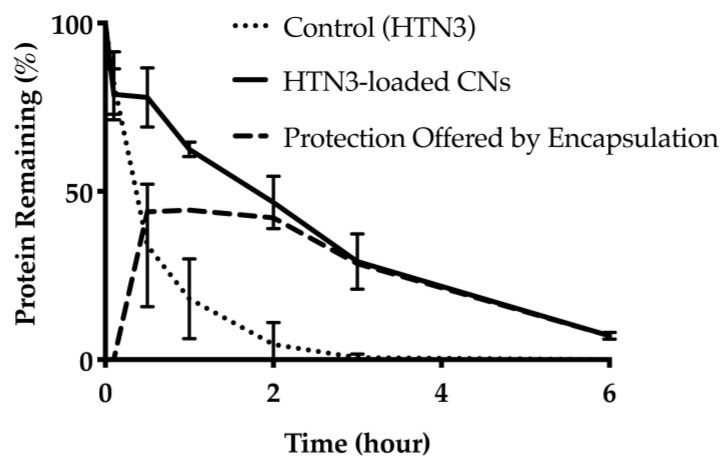
Protein degradation over time for free HTN3 and HTN3-loaded into CNs. For free HTN3, most protein was degraded in 2 h. At the same time point, about half of HTN3 was still intact in the CNs delivery system. The dashed line represents the degree of protection offered through CNs encapsulation.

To assess the protection offered through encapsulation against enzymatic degradation in the oral cavity, a protein degradation study was conducted in diluted human saliva to compare the degradation kinetics of free HTN3 and HTN3 loaded CNs. The degradation over time was quantified for both free HTN3 and HTN3 loaded CNs (Figure 5 and Table S1). For free HTN3, only 6% ± 5% of the free HTN3 remained after 2 h, whereas 47% ± 8% of HTN3 was intact when it was encapsulated in the CNs delivery system.

#### 3.1.6. Biofilm Formation

**Figure 6:**
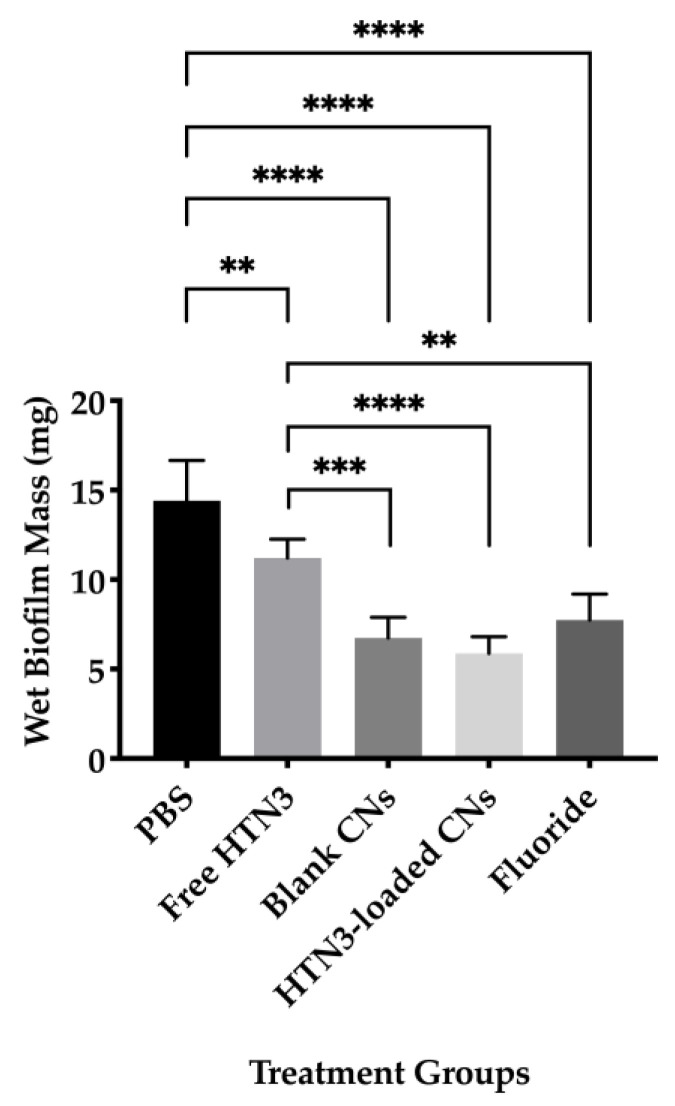
Wet biofilm masses for four treatment groups were compared against the control group PBS. All treatment groups led to significantly lower biofilm mass than PBS. Fluoride, blank, and histatin encapsulated CNs performed significantly better than free HTN3 in minimizing biofilm formation, but no significant difference in biofilm mass was observed between these treatment groups (** *p* ≤ 0.01, *** *p* ≤ 0.001, **** *p* ≤ 0.0001).

Wet biofilm mass was measured to test the effectiveness of four different treatment groups including 7.4 μM HTN3, 0.1% *w/v* unloaded CNs, 7.4 μM HTN3 in CNs, and the gold standard 12,300 ppm fluoride solution. These groups were compared against PBS as a control group. The control group had an average wet biofilm mass of 15 ± 2 mg, while the mass was 12 ± 1 mg for HTN3, 8 ± 2 mg for fluoride, 7 ± 1 mg for unloaded CNs, and 6 ± 1 for HTN3-loaded CNs (Figure 6).

**Figure 7:**
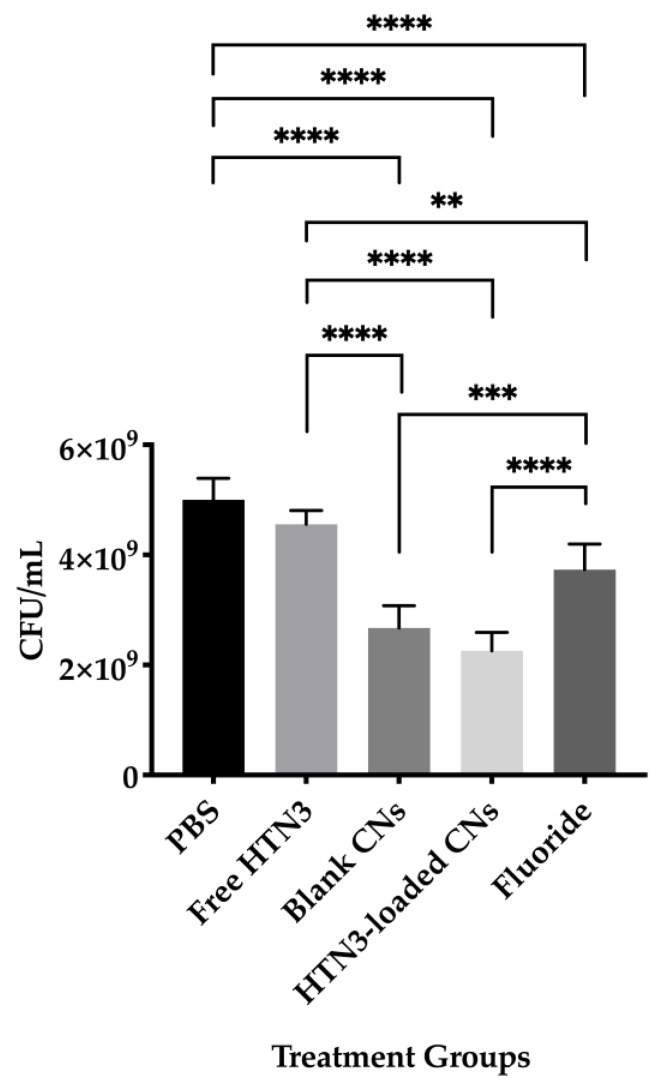
Bacterial viability for the four treatment groups were compared against the control group treated with PBS only. While free HTN3 did not control bacterial growth, fluoride treatment significantly decreased bacterial growth. Blank and HTN3-loaded CNs were the most effective at decreasing bacterial population but are insignificantly different from each other (** *p* ≤ 0.01, *** *p* ≤ 0.001, **** *p* ≤ 0.0001).

Bacterial cell viability was also evaluated and presented in Figure 7. The PBS control led to 5.0 × 10^9^ ± 0.4 × 10^8^ CFU/mL of viable bacteria, while free HTN3 had 4.6 × 10^9^ ± 0.3 × 10^9^ CFU/mL, fluoride had 3.8 × 10^9^ ± 0.5 × 10^9^ CFU/mL, unloaded CNs had 2.7 × 10^9^ ± 0.4 × 10^9^ CFU/mL, and HTN3-loaded CNs had 2.3 × 10^9^ ± 0.3 × 10^9^ CFU/mL. 

## 4. Discussion

Salivary proteins such as statherin and HTN have demonstrated several functions that are directly related to the inhibition of dental caries. They inhibit enamel demineralization, promote enamel remineralization, and modulate growth of *S. mutans* [12,41]. However, given the oral cavity can be hostile to proteins due to the high proteolytic activity, these proteins cannot be readily applied as therapeutics for protein-mediated homeostasis. In this study, we produced pH-sensitive CNs, which are able to encapsulate our protein of interest, HTN3, and release it selectively under acidic conditions. The observed pH-sensitivity is important because major oral complications, such as dental caries and dental erosion, occur under acidified conditions. The encapsulation also prolonged the lifetime of HTN3 inside 10-fold diluted human saliva.

The chitosan to TPP mass ratio was first studied systematically to optimize the formulation. At low ratios of chitosan to TPP, the particles were unstable, due to charge neutralization, resulting in visible sedimentation. They became stable as the mass ratio exceeded values ranging from 2:1 to 5:1, depending on the form of chitosan. In general, the smallest diameters and lowest PDI values were observed as a cation:anion molar ratio of about 2:1. As the ratio was further increased, the Z-average diameter increased, which can perhaps be attributed to more chitosan per particle or aggregation of the particles. For the same reasons, the PDI also followed a similar trend. For the zeta potential, a steady increase was observed with increasing chitosan to TPP ratios, since chitosan is positively charged. The findings are in agreement with many studies in the literature [42,43].

The optimal formulation was selected based on particle diameter, PDI, and zeta potential. Ideally, it is best to have a formulation with a sub-200 nm diameter nanoparticles, with a relatively low PDI and a high zeta potential. Therefore, the ultra-low MW chitosan formulation at a ratio of 6.94:1 (chitosan to TPP) was chosen as it has the smallest particle diameter of 144 ± 6 nm, with the lowest PDI of 0.15 ± 0.04 and a zeta potential of 18 ± 4 mV. Through a colloidal stability study by time-course of particle size and PDI measurements by DLS, we found that these nanoparticles maintain their average diameter and PDI when the zeta potential is above 15 mV. These optimized CNs were visualized by TEM and the average particle diameter estimated from the TEM images was in good agreement with the average particle diameter obtained from DLS. Since HTN3 is a low MW salivary protein, its encapsulation in the CNs did not significantly affect the particle diameter. As shown by the TEM images, there were no significant differences in size or dispersity between the unloaded CNs and those with different HTN3 loading ratios.

The pH-responsive properties of the optimized CNs were also investigated. The extent of swelling under different pH conditions was studied. The highest degree of swelling was observed at pH 3 and the CNs remained unchanged at pH 6.3. The results also demonstrated that the degree of swelling depended on the acidity of the suspension. The swelling response elicited by the pH stimulus was rapid, suggesting that CNs were able to quickly respond to pH changes. The versatility of this pH-responsive property was further examined through pH-cycling between 6.3 and 4, and the results suggested the CNs delivery system was capable of swelling selectively at acidic pH and reversed the swelling process upon removal of the pH stimulus.

The encapsulation loading efficiency of HTN3 was studied at 2%, 5%, and 10% *w/w* loading ratios of HTN3 to chitosan. The absence of unloaded protein suggested that HTN3 was quantitatively encapsulated in the delivery system at all ratios, which is beneficial, as the loading can therefore be tuned. Cumulative release studies at different pH values including 3, 4, 5, and 6.8 were also conducted to assess the extent of release of encapsulated HTN3 from the delivery system. These values were chosen because they reflect the cariogenic conditions promoting different oral diseases. For instance, dental caries initiate at pH 5 and dental erosion occurs at pH 3. In accordance with the demonstration of the pH-responsive property shown previously, the extent of HTN3 release was proportionate to the acidity of the environment. A maximum cumulative release of 84% ± 7% was achieved at pH 3 over 210 min, while a minimum release of 2% ± 2% was observed at pH 6.8 over the same time period. To better mimic the pH changes in the oral cavity throughout the day, pH-cycling release was performed between pH 6.8 and 4. HTN3-loaded CNs were able to selectively release encapsulated HTN3 at pH 4, and retain the HTN3 at the salivary pH of 6.8. The ability to selectively release HTN3 under acidic conditions is crucial because throughout the day the salivary pH fluctuates many times from the consumption of food or beverages [23,44]. As saliva becomes acidic following carbohydrate intake, the drop in pH could trigger the release of HTN3, which promotes oral homeostasis by inhibiting the demineralization process. Salivary pH would gradually recover to its physiological value due to the buffer capacity of saliva, which would halt HTN3 release from the CNs. Further release of HTN3 would require another sugar challenge, which would drop salivary pH.

Salivary proteins are susceptible to the high proteolytic activity in the oral cavity, which prevent them from being used as potential therapeutics on their own. To evaluate the protection offered through CN encapsulation against enzymatic degradation, a protein degradation study was performed in 10-fold diluted human saliva. Saliva was diluted to better capture the degradation kinetics of proteins, since at the original concentration the degradation would have happened too rapidly to allow easy measurement of the process. Only 6% ± 5% of the free HTN3 remained at the 2-h mark. At the same time point, 47% ± 8% of HTN3 was still present in the CN delivery system. At the 6-h mark, only 7% ± 1% of HTN3 remained in the delivery system, which could be due to the potential breakdown of the CNs by other salivary proteins. Nevertheless, the delivery system increased the protein survival time significantly. The increase in survival time is important because it allows less frequent administration of the formulation.

Ultimately, a biofilm model was applied to evaluate the therapeutic effect of HTN3-loaded CNs against *S. mutans* biofilm formation on hydroxyapatite discs. *S. mutans* was chosen because it is a major contributor responsible for the initiation and development of tooth decay [23,45]. It metabolizes sucrose to produce sticky polysaccharides that allow the bacteria to aggregate and adhere to the tooth enamel, forming a biofilm. The biofilm, together with frequent sugar intake promotes fermentation of dietary sugar into acidic products. Persistence of the resulting acidic conditions favours the proliferation of acidogenic bacteria. The low pH environment in the biofilm initiates the dental caries process [23,46]. Hydroxyapatite is a mineral composed of calcium phosphate, which greatly resembles human hard tissues including bone and tooth enamel in composition and morphology [47]. It is also the most stable calcium phosphate mineral under physiological conditions [48]. Therefore it has been extensively used in oral applications, such as pellicle formation and biofilm formation [49,50].

Two key factors used to evaluate biofilm growth are biofilm mass and bacterial cell viability. Effective treatment should result in low biofilm mass and reduced bacterial cell viability. For biofilm mass measurements, based on the ANOVA analysis and Tukey’s multiple comparisons test, all four treatment groups resulted in significantly reduced biofilm mass compared to the control group. Fluoride together with unloaded and HTN3-loaded CNs were significantly more effective than free HTN3. However, no significant difference was observed between the HTN3-loaded CNs and unloaded CNs. Both of these treatments successfully lowered the biofilm mass by at least half. Bacterial cell viability was also determined for all treatment groups. Free HTN3 was not effective at reducing bacterial cell viability and did not perform better than the control group. Fluoride, unloaded, and HTN3-loaded CNs significantly reduced cell viability when compared against the control group. Fluoride was significantly more effective when compared against HTN3. Unloaded and HTN3-loaded CNs were the most competent treatment groups. They reduced cell viability in half, but their performance in reducing bacterial population were not significantly different from one another. While these results suggest that CNs alone can potentially control bacterial population growth, further studies that encapsulate greater HTN3 concentrations within CNs, or reduce the amount of CNs while maintaining HTN3 concentrations are required. In addition, CNs are known to exhibit antimicrobial effects against *S. mutans* [51]. Another possible explanation would be CNs alone already prevented the initiation of demineralization, therefore there was no steep drop in pH to trigger HTN3 release from the CNs.

## 5. Conclusions

In conclusion, the results of this study demonstrated the pH-responsive properties of the CNs. The CNs were also able to offer protection against enzymatic degradation in comparison to free HTN3. While HTN3-loaded CNs had successfully reduced biofilm growth as reflected by reduced biofilm mass and bacterial cell viability, these particles did not significantly outperform unloaded CNs. Future studies should include use of the biofilm model to characterize the contribution of HTN3 within CNs and CNs alone in controlling bacterial population. Nonetheless, this work has shown that CNs can be used as a protein carrier for oral applications, especially for complications involving acidic environments. This delivery system can also be applied to encapsulate other salivary proteins for oral delivery. The ultimate goal of studying protein encapsulation within CNs is to integrate multifunctional salivary proteins into daily dental hygiene products like toothpaste and mouthwash to provide a preventative approach to address dental caries. Altogether, our results show that a pH-sensitive delivery system that can release salivary proteins under oral conditions can be achieved by utilizing a biodegradable, biocompatible, and naturally derived polymer like chitosan.

## Data Availability

The data presented in this study are available on request from the corresponding author.

## Supplementary Materials

The following are available online at https://www.mdpi.com/article/10.3390/nano11041028/s1, Figure S1: Volume distribution of the ultra-low MW CNs at chitosan to TPP mass ratio of 6.94. There is a small batch to batch variability; Figure S2: CNs were stored at 4 °C, and three parameters including (a) Z-average diameter, (b) PDI, and (c) Zeta potential were measured by DLS routinely over the course of 60 days. The consistency of obtained data suggest the nanoparticles are stable; Figure S3: TEM images of (a) blank chitosan nanoparticles, (b) HTN3 loaded chitosan nanoparticles with a 2% *w/w* loading ratio, (c) HTN3 loaded chitosan nanoparticles with a 5% *w/w* loading ratio, and (d) HTN3 loaded chitosan nanoparticles with a 10% *w/w* loading ratio. No significant variance in size or dispersity was observed among blank chitosan nanoparticles and those with different loading ratios; Table S1: Protein degradation over time for free HTN3 and HTN3-loaded into CNs.
