# Supplementary material for: pH-Sensitive Chitosan Nanoparticles for Salivary Protein Delivery
Source: Nanomaterials (Basel). 2021 Apr 17;11(4):1028. doi: 10.3390/nano11041028 (PMC8073935; doi:10.3390/nano11041028)
Supplement: Supplementary file 1 [file nanomaterials-11-01028-s001.zip › nanomaterials-1167974-supplementary.pdf]

Supporting information for:

pH-Sensitive Chitosan Nanoparticles for Salivary Protein Delivery

Yi Zhu [1], Lina Marin [2], Yizhi Xiao [3], Elizabeth R. Gillies [1,4] and Walter L. Siqueira [2,*]

[1] School of Biomedical Engineering, The University of Western Ontario, 1151 Richmond Street, London, ON N6A 3K7, Canada

[2] College of Dentistry, University of Saskatchewan, 105 Wiggins Rd, Saskatoon, SK S7N 5E4, Canada

[3] Schulich Medicine and Dentistry, The University of Western Ontario, 1151 Richmond Street, London, ON N6A 5C1, Canada

[4] Department of Chemistry, Department of Chemical and Biochemical Engineering, The University of Western Ontario, 115 Richmond Street, London, ON, N6A 5B7

* Correspondence: walter.siqueira@usask.ca

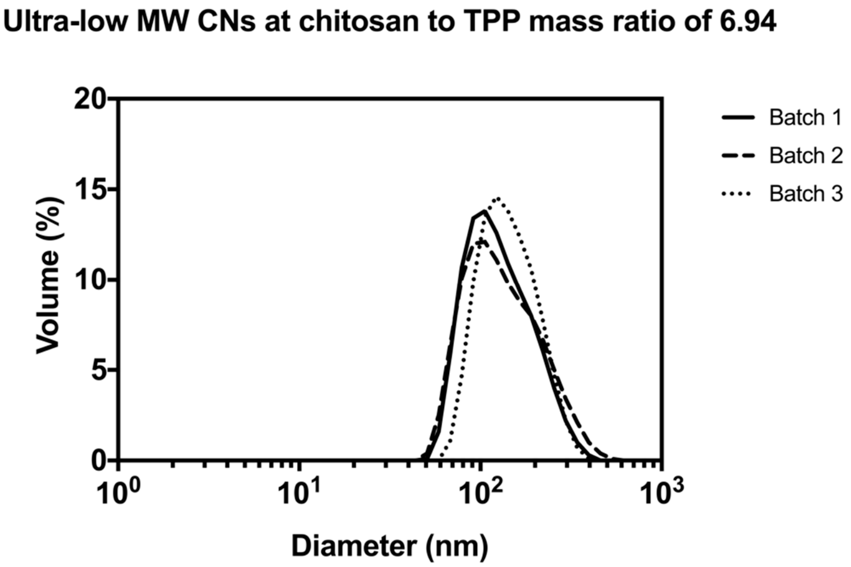

Figure S1. Volume distribution of the ultra-low MW CNs at chitosan to TPP mass ratio of 6.94. There is a small batch to batch variability.

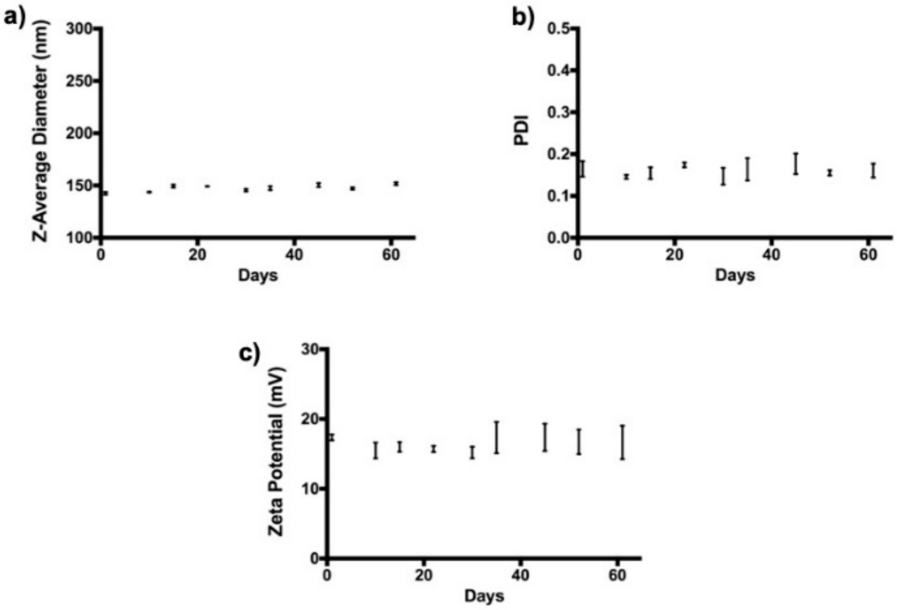

Figure S2. CNs were stored at 4°C, and three parameters including (a) Z-average diameter, (b) PDI, and (c) Zeta potential were measured by DLS routinely over the course of 60 days. The consistency of obtained data suggest the nanoparticles are stable.

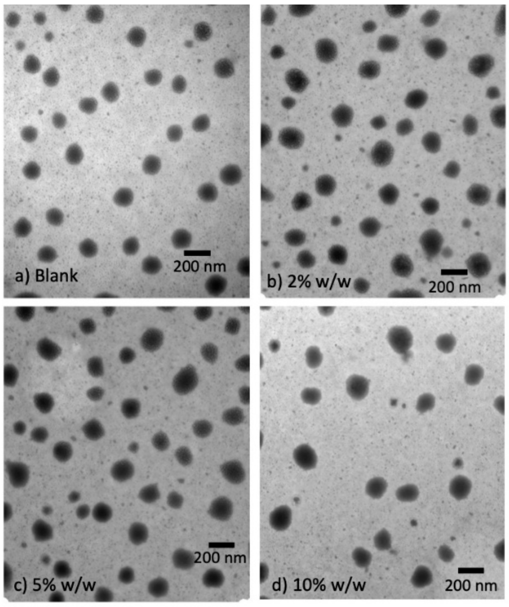

Figure S3. TEM images of (a) blank chitosan nanoparticles, (b) HTN3 loaded chitosan nanoparticles with a 2% w/w loading ratio, (c) HTN3 loaded chitosan nanoparticles with a 5% w/w loading ratio, and (d) HTN3 loaded chitosan nanoparticles with a 10% w/w loading ratio. No significant variance in size or dispersity was observed among blank chitosan nanoparticles and those with different loading ratios.

Table S1. Protein degradation over time for free HTN3 and HTN3-loaded into CNs.

	Remaining HTN3 (%)	
**Time (h)**	**Free HTN3**	**HTN3-loaded CNs**
0	100	100
0.1	82 ± 9	79 ± 8
0.5	34 ± 18	78 ± 9
1	18 ± 12	63 ± 2
2	6 ± 5	47 ± 8
3	1 ± 1	29 ± 8
6	0	7 ± 1
